# SFPQ Promotes Hepatocellular Carcinoma Progression by Affecting GTF2H3 Splicing Variants

**DOI:** 10.3390/cancers18203258

**Published:** 2026-10-09

**Authors:** Qin Tang, Siyuan Chen, Xueying Chen, Jian Gao, Zhihang Zhou

**Affiliations:** Department of Gastroenterology, The Second Affiliated Hospital of Chongqing Medical University, Chongqing 400010, China; 305254@hospital.cqmu.edu.cn (Q.T.); chensiyuan@cqmu.edu.cn (S.C.);

**Keywords:** HCC, alternative splicing, SFPQ, GTF2H3, prognostic biomarker

## Abstract

**Background:** Hepatocellular carcinoma (HCC), the predominant type of liver cancer, exhibits a high mortality rate due to unclear molecular mechanisms and limited biomarkers. Accumulating evidence indicates that splicing factors (SFs) can promote tumor cell proliferation, invasion, metastasis, and drug resistance by altering the splicing patterns of target genes. However, the regulatory mechanisms underlying SF-mediated alternative splicing (AS) are highly intricate, and the specific functions and pathways of SFs in HCC initiation and progression remain to be further elucidated. **Methods:** First, Cox regression and LASSO regression analyses of splicing factors (SFs) were performed using the TCGA database, followed by validation using the ICGC dataset. The prognostic value of SFPQ was assessed with the Kaplan–Meier plotter. Next, the potential association of SFPQ with the immune microenvironment of HCC was explored using TIMER and CIBERSORT. In addition, we screened AS events associated with SFPQ expression in TCGA-LIHC, conducted GO/KEGG pathway enrichment analyses, and investigated the correlation between GTF2H3 percent spliced in index (PSI) values and clinicopathological features in HCC patients. Finally, the effect of targeting the exon 10- or exon 11-containing variants of GTF2H3 on HCC was examined at the cellular level. **Results:** LASSO regression analysis identified five key SFs significantly associated with HCC prognosis, including SFPQ, HTRA2, DAZAP1, PCBP2, and YBX1. Among these, SFPQ was determined as the most critical SF influencing HCC survival, and its overexpression was positively correlated with HCC progression. GO and KEGG analyses revealed that the pathways potentially associated with SFPQ include metabolic pathways, oxidative phosphorylation, mRNA surveillance pathway, and the PPAR signaling pathway. Furthermore, SFPQ expression was associated with the exclusion of exons 10 and 11 of the *GTF2H3* gene. Computational analysis also suggested a potential link between SFPQ and immune cell infiltration. Targeting the exon 11-containing variant of GTF2H3 significantly reduced cell viability and migration of HCC cells. **Conclusion:** SFPQ expression is elevated in HCC and correlates with tumor aggressiveness and poor patient prognosis. Our findings suggest that SFPQ may influence HCC progression through its association with the GTF2H3 exon 10/11 ME event and the immune microenvironment. However, further experimental validation is required to confirm these mechanisms. Our findings suggest that SFPQ may serve as a promising biomarker and therapeutic candidate for HCC.

## Simple Summary

Hepatocellular carcinoma (HCC) is a highly lethal form of liver cancer, and new biomarkers are urgently needed to improve patient outcomes. In this study, we analyzed large public databases and identified the RNA-binding protein SFPQ as a key factor associated with poor survival in HCC patients. Our computational analysis suggests that SFPQ expression is linked to immune cell infiltration and may influence the alternative splicing of the *GTF2H3* gene, specifically the mutually exclusive exons 10 and 11. Functional experiments in HCC cell lines further revealed that targeting the exon 11-containing variant of GTF2H3 significantly reduced cell viability and migration. Although further experimental validation is required, these findings suggest that SFPQ and the GTF2H3 splice variant may serve as potential prognostic biomarkers and therapeutic targets for HCC.

## 1. Introduction

Liver cancer is an aggressive malignancy characterized by high incidence and mortality rates. According to the GLOBOCAN 2020 database, approximately 905,000 new cases and 830,000 deaths were reported globally in 2020 [1]. Projections indicate that by 2040, the number of new diagnoses will reach 1.4 million, with approximately 1.3 million deaths [2]. Primary liver cancer mainly comprises hepatocellular carcinoma (HCC), intrahepatic cholangiocarcinoma, and mixed hepatocellular carcinoma, of which HCC accounts for 75–85% of cases. Despite improvements in therapeutic approaches—such as precise liver resection, radiotherapy, transarterial therapy, chemotherapy, targeted therapy, and immunotherapy—the 5-year overall survival rate for HCC remains exceedingly low [3]. Unclear molecular mechanisms and the lack of disease-specific biomarkers severely hinder the diagnosis and treatment of HCC. Therefore, novel therapeutic targets are urgently needed to improve the prognosis and clinical management of this malignancy. Alternative splicing (AS) serves as a fundamental mechanism for generating transcriptomic and proteomic complexity and functional diversity in eukaryotes. Beyond its essential roles in physiological processes such as hematopoiesis [4], brain development [5], and muscle function [6], AS also contributes substantially to the pathogenesis of numerous systemic disorders and malignancies [7]. The regulatory information directing splicing events is generally considered to be encoded within cis-acting elements located in exons and introns. These elements recruit sequence-specific RNA-binding proteins that either activate or inhibit the use of adjacent splice sites [4,5]. The utilization of multiple splice sites allows for the generation of distinct mRNA isoforms from a single precursor RNA, thereby substantially increasing protein diversity. Aberrant splicing site recognition due to mutations or dysregulation of splicing factors can produce improperly processed mRNA variants or pathogenic isoforms, which significantly drive tumor progression and malignancy [8]. Existing evidence indicates that AS modulates protein diversity and cellular phenotypes by generating multiple mature mRNA isoforms with distinct functions through the selective splicing of precursor mRNAs [9,10]. In breast cancer, aberrant expression of alternative splicing factors—such as pre-mRNA processing factor 8 (PRPF8)—can lead to intron retention and an increase in abnormal splicing variants, thereby promoting apoptosis and affecting protein metabolism, mitosis, and proteasome function [11]. In pancreatic cancer, overexpression of the splicing factors PRPF8 and RBMX is significantly associated with specific splicing events, including exon skipping and intron retention [12]. In HCC, loss of Serine/Arginine-Rich Splicing Factor 3 (SRSF3) induces insulin-like growth factor II (IGF2) expression and, by altering INSR splicing, enables IGF2 signaling to occur via the insulin receptor (IR)-A in hepatocytes [13]. Research has shown that alternative splicing factors (SFs) can promote tumor cell proliferation, invasion, metastasis, and drug resistance by altering the splicing patterns of target genes. In HCC, aberrant expression of SFs leads to the generation of oncogenic or tumor-suppressive splice variants of specific genes, representing a core mechanism of alternative splicing function [14,15,16]. These factors exert their effects within HCC cells by converting alternative splicing products into pro-tumor isoforms, thereby regulating cell proliferation, invasion, and metastasis [17,18,19]. Although these observations suggest that abnormal splicing plays a significant role in HCC development, the underlying mechanisms require further investigation.

Splicing Factor Proline and Glutamine Rich (SFPQ), also known as PSF or PPP1R140, is an RNA-binding protein located on chromosome 1 (1p37.3) and belongs to the family of protein phosphatase 1 regulatory subunits containing RNA-binding moils [20]. As a core regulator of pre-mRNA processing, SFPQ recognizes cis-acting regulatory elements—including splicing enhancers or silencers in exons or introns—and recruits sequence-specific factors to modulate alternative splicing events [21,22]. Notably, in the context of tumor immunity, SFPQ has been identified as a key modulator of Th1 cell function. SFPQ binds to pre-mRNA of the transcription factor IRF1, driving its alternative splicing to produce a truncated isoform (IRF1Δ7). This alternative isoform sequesters full-length IRF1, inhibiting its transcriptional activity and ultimately impairing IFNγ secretion and Th1-mediated anti-tumor immune responses [23]. Although this mechanism has been validated in some tumor models (such as colorectal cancer and breast cancer), its relevance to HCC remains to be elucidated. Beyond its canonical role in mRNA splicing, SFPQ is also involved transcriptional regulation and DNA damage repair [24]. Existing studies have revealed that SFPQ drives HCC progression through multiple mechanisms, including metabolic reprogramming (e.g., fatty acid synthesis) [25], signaling pathway activation (e.g., IL-6/STAT3 and necroptosis) [26], RNA regulation, and modulation of the immune microenvironment [23,27]. Clinically, SFPQ overexpression correlates with poor prognosis in HCC patients, highlighting its potential as a prognostic biomarker and therapeutic target. Functional assays have shown that silencing SFPQ significantly inhibits the proliferation and migration of HCC cell lines (Huh-7 and Hep3B) [28]. Mechanistically, GO and KEGG enrichment analyses of SFPQ-associated gene modules reveal significant enrichment in RNA splicing-related processes, including ester exchange-mediated splicing, spliceosome assembly, and mRNA processing. These findings suggest that SFPQ may drive HCC progression by dysregulating alternative splicing pathways [28]. However, the specific downstream targets and context-dependent regulatory mechanisms remain unclear. Therefore, the present study aimed to investigate how the alternative splicing factor SFPQ regulates alternative splicing events to promote HCC progression.

Therefore, we first performed survival and differential expression analyses of splicing factors using The Cancer Genome Atlas (TCGA) data and identified SFPQ as the splicing factor with the most significant impact on patient survival. This finding was subsequently validated using the International Cancer Genome Consortium (ICGC) dataset. The prognostic value of SFPQ was further evaluated via the Kaplan–Meier Plotter database, and its role in the HCC immune microenvironment was explored using TIMER and CIBERSORT. To investigate potential pathogenic mechanisms of SFPQ in HCC, SFPQ expression-associated alternative splicing events (ASEs) in the TCGA LIHC cohort were analyzed, followed by Gene Ontology (GO) and Kyoto Encyclopedia of Genes and Genomes (KEGG) pathway enrichment analyses on the relevant target genes. Our results indicated that SFPQ may promote HCC progression by regulating exons 10 and 11, thereby influencing exon exclusion events in GTF2H3. Subsequently, the role of SFPQ in modulating the HCC microenvironment was explored using data from the Tumor Immune Microenvironment Resource (TIMER) and the TCGA LIHC dataset. Finally, biological function of GTF2H3 in MHCC97H and SNU449 cells was investigated by knocking down its expression (specifically exon 10 or exon 11) using siRNA. Our study demonstrates that SFPQ is a splicing-associated factor linked to survival in HCC, and its overexpression is associated with aggressive clinical and pathological features and poor prognosis. SFPQ contributes to its oncogenic role by influencing the functional effects of specific splice variants (e.g., transcripts containing GTF2H3 ME), as well as by modulating immune cell infiltration and immune checkpoint expression. These findings suggest that SFPQ holds promise as a biomarker and therapeutic target for HCC.

## 2. Materials and Methods

### 2.1. Data Extraction Process

We used alternative splicing factor data from pliceAid 2, which contained 71 splicing factors (SFs) The RNA-Seq and relevant clinical information of TCGA-LIHC cohort were obtained from TCGA. The RNA-seq data included 371 tumor tissues and 50 adjacent tissues, and 50 of them were matched specimens. The ASEs of HCC patient data were downloaded from TCGA-spliceSeq, which included seven types of ASEs and 259 patients. The percent spliced in index (PSI), ranging from 0 to 1, was frequently used to assess the proportion of a specific gene transcript exhibiting a particular splicing pattern. To generate the most reliable set of ASEs, missing values in the data were imputed using the k-nearest neighbors algorithm from the R package (V 4.2.1). The correlation between SFPQ expression and GTF2H3 PSI was assessed using Pearson correlation analysis. To control for potential confounders, partial correlation analysis was performed using the ppcor package in R, adjusting for tumor stage and grade.

### 2.2. Survival and Differential Analysis

Univariate and multivariate Cox regression analyses were employed to evaluate the correlation between SFs and overall survival (OS-SFs). To ensure the validity of the Cox proportional hazards model, the proportional hazards assumption was tested using the Schoenfeld residuals test (via the cox.zph() function in R). To identify key SFs affecting HCC survival, LASSO regression analysis was conducted using R. Specifically, LASSO regression was performed with the glmnet package in R, employing 10-fold cross-validation to determine the optimal penalty parameter (lambda.min). Concurrently, we separately evaluated the relationship between the seven types of ASEs and overall survival (OS-ASEs). The R package ‘survival’ was used to assess the association between key functional gene expression and overall survival in HCC. We performed survival and differential analysis using the TCGA-LIHC cohort as our experimental dataset. Additionally, we downloaded the ICGC-LIRI-JP dataset as our validation set, which comprises 206 hepatocellular carcinoma samples and 177 adjacent normal tissue samples; 175 of these constitute paired samples.

### 2.3. SF and ASE Network Construction

Spearman correlation analysis was performed to evaluate the relationships between OS-SFs and OS-ASEs using the R package ‘corrplot’. Pairs with a correlation coefficient greater than 0.45 and a *p*-value less than 0.005 were selected as significantly associated SF–OS-ASE pairs for subsequent analysis. Finally, a network diagram was constructed using Cytoscape (version 3.9.1) to visualize the significantly co-expressed OS-SFs and OS-ASEs.

### 2.4. GO and KEGG Analysis

Alternative splicing events associated with SFPQ expression were evaluated. Subsequently, GO and KEGG enrichment analyses were performed on genes exhibiting alternative splicing events significantly correlated with SFPQ expression using the DAVID database (https://davidbioinformatics.nih.gov/).

### 2.5. Immune Cell Infiltration Analysis

Based on the TCGA-LICH cohort, the relative proportions of tumor-infiltrating immune cells (TIICs) were calculated using CIBERSORT (version 1.0; https://cibersort.stanford.edu/, accessed on 25 August 2026). For the correlation analysis of immune cell infiltration, the Benjamini–Hochberg method was used to adjust *p*-values for multiple testing. HCC specimens were stratified into high-SFPQ and low-SFPQ groups according to the median SFPQ expression level, and the relative abundance of TIICs was compared between the two groups. Association analyses between SFPQ expression and various immune cell types as well as their respective gene markers were performed using the TIMER database (http://timer.cistrome.org). Additionally, correlations between SFPQ expression levels and immune checkpoint inhibitors, chemokines, and chemokine receptors in HCC were evaluated through the TISIDB database (http://cis.hku.hk/TISIDB/, accessed on 25 August 2026).

### 2.6. Cell Culture and Transfection

The human hepatocellular carcinoma cell lines Huh7, PLC/PRF/5, HepG2, SK-Hep1, HEP3B, and MHCC97H, as well as the normal liver cell line THLE3, were all purchased from Servicebio Technology Company (Wuhan, China). Cells were cultured in DMEM (MHCC97H) with 10% fetal bovine serum (FBS, Azaood, Dover, DE, USA) or RPMI-1640 (SNU449) with 10% FBS (Azaood, Dover, DE, USA) at 37 °C in a 5% CO_2_ atmosphere. Before transfection, cells were cultured to approximately 50% confluence in 6-well plates. We used EndoFectin™ Transfection Reagents (GeneCopoeia, Rockville, MD, USA) to transfect the small interfering RNA (siRNA) (Tsingke, Beijing, China). To ensure the specificity of targeting the mutually exclusive exons of GTF2H3, three independent siRNAs were designed for each target region. As detailed in Supplementary Table S1, these siRNAs were designed to target either the internal sequences of exon 10 or exon 11 or the specific exon–exon junctions (e.g., exon 10/11 and exon 11/12 boundaries). After transfection with siRNA for 48 h, real-time quantitative polymerase chain reaction (RT-PCR) was performed to examine the transfection efficiency. The sequences of siRNA are shown in Table 1.

### 2.7. RNA Extraction and RT-PCR

Total RNA was extracted using the SPARK easy Cell RNA Kit (Sparkjade, Qingdao, Shandong, China). Reverse transcription was performed with the SPARK script II All-in-one RT SuperMix for qPCR (with gDNA Eraser) (Sparkjade). RT-qPCR was carried out using 2 × SYBR Green qPCR Mix (Sparkjade), with β-actin as the internal reference for relative expression normalization. The primer sequences are listed in Table 2. Relative RNA expression levels were calculated using the 2^−ΔΔCt^ method.

### 2.8. Cell Viability Assay

Cell Counting Kit-8 (Sparkjade, China) assay: After interference with siRNA for 48 h, transfected cells were plated in 96-well plates at a density of 1 × 10^3^ cells/well. From the first to the fifth day, the serum-free medium of 10% CCK-8 reaction solution was used to replace the original medium and incubated for 2 h. The light absorbance was quantified at 450 nm (OD-450).

### 2.9. Cell Migration

The upper chambers were purchased from Labselect (Beijing, China). The brief procedure is as follows: To assess tumor cell migration capacity, 6 × 10^4^ cells were seeded into the uncoated upper chamber. In a 24-well plate, the upper wells were filled with 200 µL of serum-free medium, while the lower wells were received 800 µL of medium containing 20% FBS. Cells were cultured for 24 h. At the observation time point, the cells adhering to the membrane surface of the upper chamber were removed using a cotton swab. The migrating cells were fixed with methanol and stained with 0.1% crystal violet. Cell counts were performed in five distinct areas under a microscope.

All functional assays (RT-qPCR, CCK-8, and migration) were performed with at least three independent biological replicates (*n* ≥ 3).

### 2.10. Statistical Analysis

All statistical analyses were conducted using R software (version 4.2.1) and GraphPad Prism (version 9.0.2; GraphPad Software, Inc., San Diego, CA, USA). Comparisons between two groups of continuous variables were performed using Student’s *t*-test. Nonparametric tests included the Kruskal–Wallis test and the Wilcoxon test. Potential prognostic factors were identified by univariate Cox analysis and LASSO analysis. For comparisons between multiple groups and a single control, one-way ANOVA followed by Dunnett’s test was applied. For multi-day CCK-8 assays, two-way ANOVA was used. In the CCK-8 experiments, statistical significance markers in the figures indicate the results of comparisons at the final time point. A *p*-value < 0.05 was considered statistically significant.

## 3. Result

### 3.1. Identification of Survival-Related Alternative Splicing Factors in HCC

**Figure 1:**
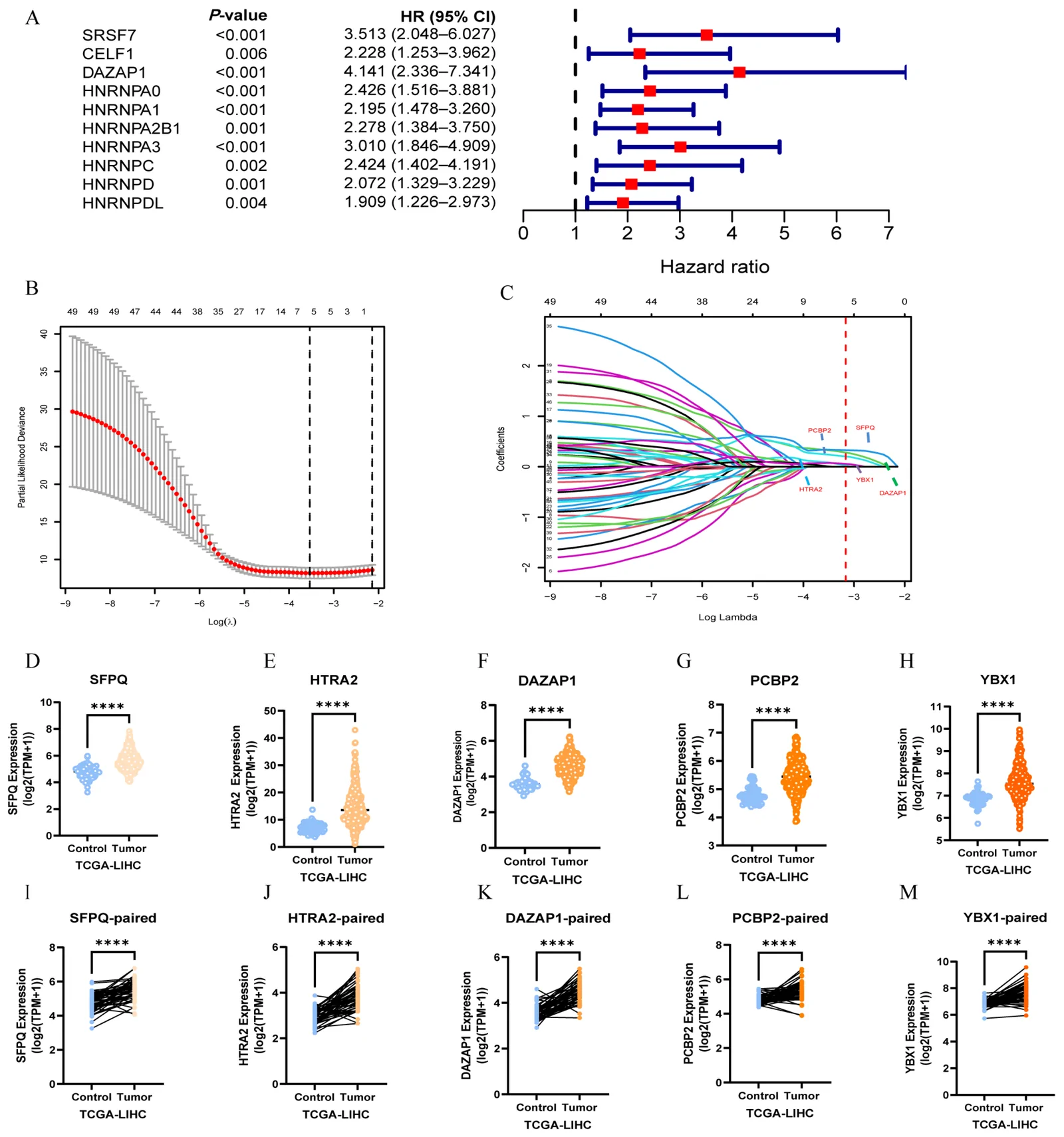
Identification of survival-related alternative splicing factors in HCC. (**A**) Univariate analysis of SF expression and overall survival(OS). (**B**) Screening of prognosis-associated alternative splicing factors via LASSO regression analysis in the TCGA-LIHC cohort. The partial likelihood deviance was plotted against log (λ). The left dashed vertical line indicates the optimal λ value (λ.min), which resulted in the selection of 5 candidate factors. The right dashed line represents the λ.1se value. The numbers along the top axis indicate the number of non-zero coefficients (selected factors) at the correspondingλvalues. (**C**) LASSO coefficient profiles of the 5 candidate alternative splicing factors. (**D**–**H**) Based on TCGA-LIHC cohort, unpaired analyses show the mRNA expressions of these key SFs (including SFPQ, HTRA2, DAZAP1, PCBP2, and YBX1) in HCC and normal samples. (**I**–**M**) Paired analysis show the mRNA expression levels these key SFs in HCC and adjacent samples. (**** *p* < 0.0001).

A total of 71 SFs were retrieved from the spliceAid 2 database (Supplementary Table S2). Leveraging TCGA data, the transcriptomic profiles and survival outcomes of 259 HCC patients were analyzed to pinpoint SFs correlated with patient prognosis. Cox proportional hazards regression identified 50 SFs significantly linked to survival (*p* < 0.05, HR > 1) (Figure 1A; Supplementary Table S3). Subsequent LASSO regression refined this list to five pivotal SFs—SFPQ, HTRA2, DAZAP1, PCBP2, and YBX1—exhibiting robust associations with HCC outcomes (Figure 1B,C).

Expression analysis within the TCGA-LIHC cohort revealed markedly elevated mRNA levels of these five SFs in tumor tissues (*n* = 374) relative to normal counterparts (*n* = 50) (*p* < 0.001; Figure 1D–H). To substantiate these observations, the ICGC-LIRI-JP dataset served as an independent validation cohort. Consistently, SFPQ, HTRA2, DAZAP1, PCBP2, and YBX1 were significantly overexpressed in tumor specimens (*n* = 212) compared to adjacent non-tumor tissues (*n* = 177) (Figure S1A–E). Paired-sample analyses across both datasets further corroborated these upregulations (Figure 1I–M and Figure S1F–J). Collectively, these findings across multiple cohorts underscore the pronounced overexpression of these SFs in HCC relative to normal liver tissue.

### 3.2. Relationship Between the Expression of These Key SFs and Clinicopathological Characteristics in HCC Patients

**Figure 2:**
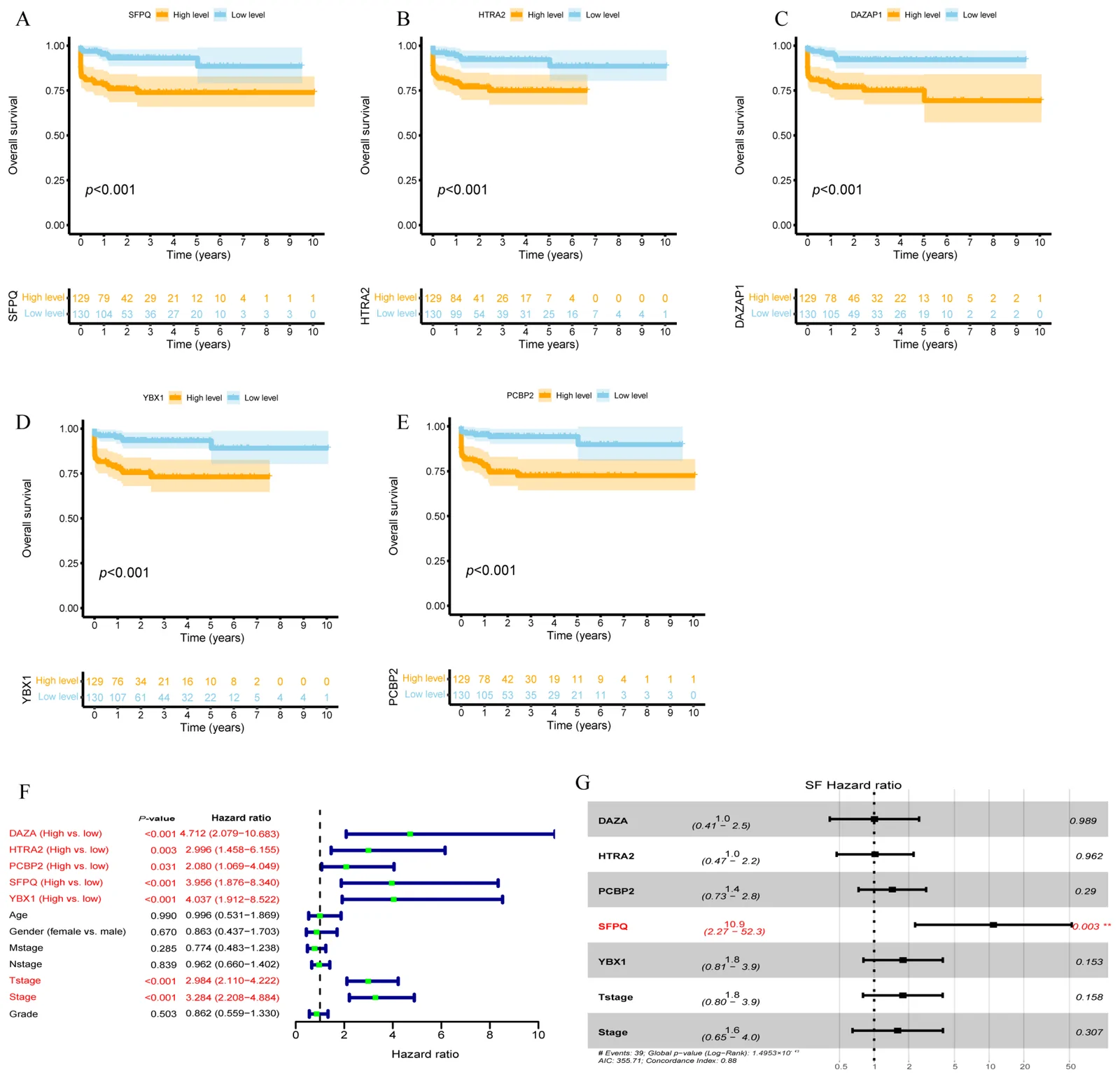
The key SFs related with an unfavorable prognosis in HCC patients. (**A**–**E**) The overall survival prognostic value of the key SFs in HCC (Kaplan–Meier plotter) in the TCGA–LIHC cohort. (**F**) Univariate analysis of the key SF expressions and clinical parameters. The dashed vertical line indicates a hazard ratio of 1. (**G**) Multivariate analysis of the key SF expressions and clinical parameters. The ** symbol indicates statistical significance at *p* < 0.01. # Detailed statistical information including the number of events, global *p*-value, Akaike Information Criterion (AIC), and Concordance Index is provided at the bottom of the figure.

We assessed the prognostic significance of these SFs in HCC using the TCGA-LIHC dataset. Upregulation of SFPQ, HTRA2, DAZAP1, PCBP2, and YBX1 was significantly associated with reduced OS (Figure 2A–E). Validation in the ICGC-LIRI-JP dataset yielded consistent results, except for YBX1 (*p* = 0.076) (Supplementary Figure S2A–E). Kaplan–Meier analyses further explored the relationship between these SFs and recurrence-free survival (RFS), progression-free survival (PFS), and disease-specific survival (DSS). Notably, only SFPQ and PCBP2 expression correlated significantly with PFS, RFS, and DSS (all *p* < 0.05) (Supplementary Figure S3).

Univariate and multivariate Cox regression analyses were conducted to assess the associations between clinical parameters (including age, sex, clinical grade, stage, M stage, N stage, and T stage) and the mRNA expression levels of SFPQ, HTRA2, DAZAP1, PCBP2, and YBX1. Univariate analysis revealed that overexpression of these five SFs was a significant predictor of poorer overall survival (OS) (*p* < 0.001, Figure 2F). Variables that reached statistical significance in the univariate analysis were subsequently entered into a multivariate Cox proportional hazards model. Notably, the multivariate analysis identified SFPQ alone as an independent prognostic factor for poor outcomes in patients with hepatocellular carcinoma (HCC) (*p* = 0.003, Figure 2G). To ensure the validity of this model, we tested the proportional hazards assumption using Schoenfeld residuals. The assumption was satisfied for SFPQ (Chisq = 3.356, *p* = 0.067, Table S4). Accordingly, given its robust independent prognostic value, SFPQ was selected as the primary molecular target for further in-depth investigation.

**Figure 3:**
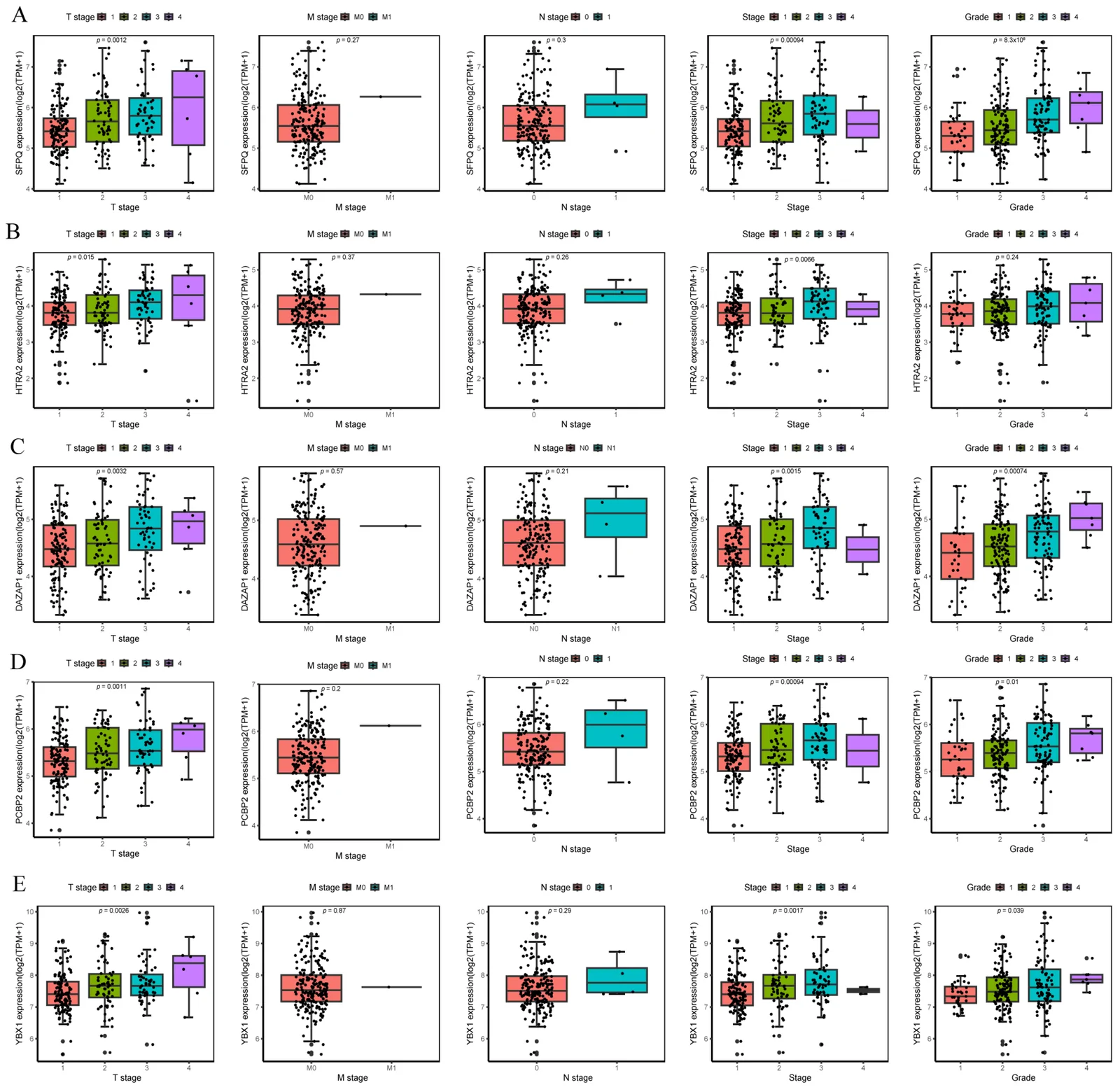
The boxplot shows the correlation between the key SFs’ expression and clinicopathological parameters, including the T stage (Kruskal–Wallis test), M stage (Wilcox test), N stage (Wilcox test), stage (Kruskal–Wallis Test), and grade (Kruskal–Wallis test). (**A**) SFPQ, (**B**) HTRA2, (**C**) DAZAP1, (**D**) PCBP2, and (**E**) YBX1.

The correlation between SF expression and clinicopathological features in HCC patients is depicted in Figure 3. Elevated levels of SFPQ, DAZAP1, PCBP2, and YBX1 were significantly associated with an advanced T stage (*p* = 0.0012, 0.0032, 0.001, and 0.0026, respectively), overall stage (*p* = 0.00094, 0.0015, 0.00094, and 0.0017, respectively), and higher histological grade (*p* = 8.3 × 10^−5^, 0.00074, 0.01, and 0.039, respectively). Although HTRA2 expression correlated with the T stage (*p* = 0.015) and overall stage (*p* = 0.0066), it showed no significant association with tumor grade (*p* = 0.24). None of the five SFs demonstrated a significant correlation with the N stage or M stage.

### 3.3. Functional Analysis of Candidate Splicing Factor SFPQ

SFPQ, as a critical splicing factor, likely exerts comprehensive influences on alternative splicing events in HCC. To elucidate the underlying mechanisms, we identified and analyzed 459 SFPQ-associated alternative splicing (AS) events, which corresponded to 385 genes (Supplementary Figure S4A). GO enrichment analyses revealed that these genes were significantly enriched in key biological processes such as the lipid metabolic process, apoptotic process, DNA damage response, mRNA processing, and DNA repair. Key cellular components (CCs) encompassed the cytoplasm, cytosol, nucleus, nucleoplasm, and mitochondrion. SFPQ-associated alternative splicing genes primarily regulated molecular functions such as protein binding, transferase activity, RNA binding, identical protein binding, and oxidoreductase activity. KEGG enrichment analysis showed metabolic pathways, oxidative phosphorylation,an mRNA surveillance pathway, and a PPAR signaling pathway. These pathways also play crucial roles in intracellular signaling pathways and metabolic processes essential for HCC development (Supplementary Figure S4B,C).

### 3.4. Relationship Between SFPQ Expression and Immune Cell Infiltration in HCC

**Figure 4:**
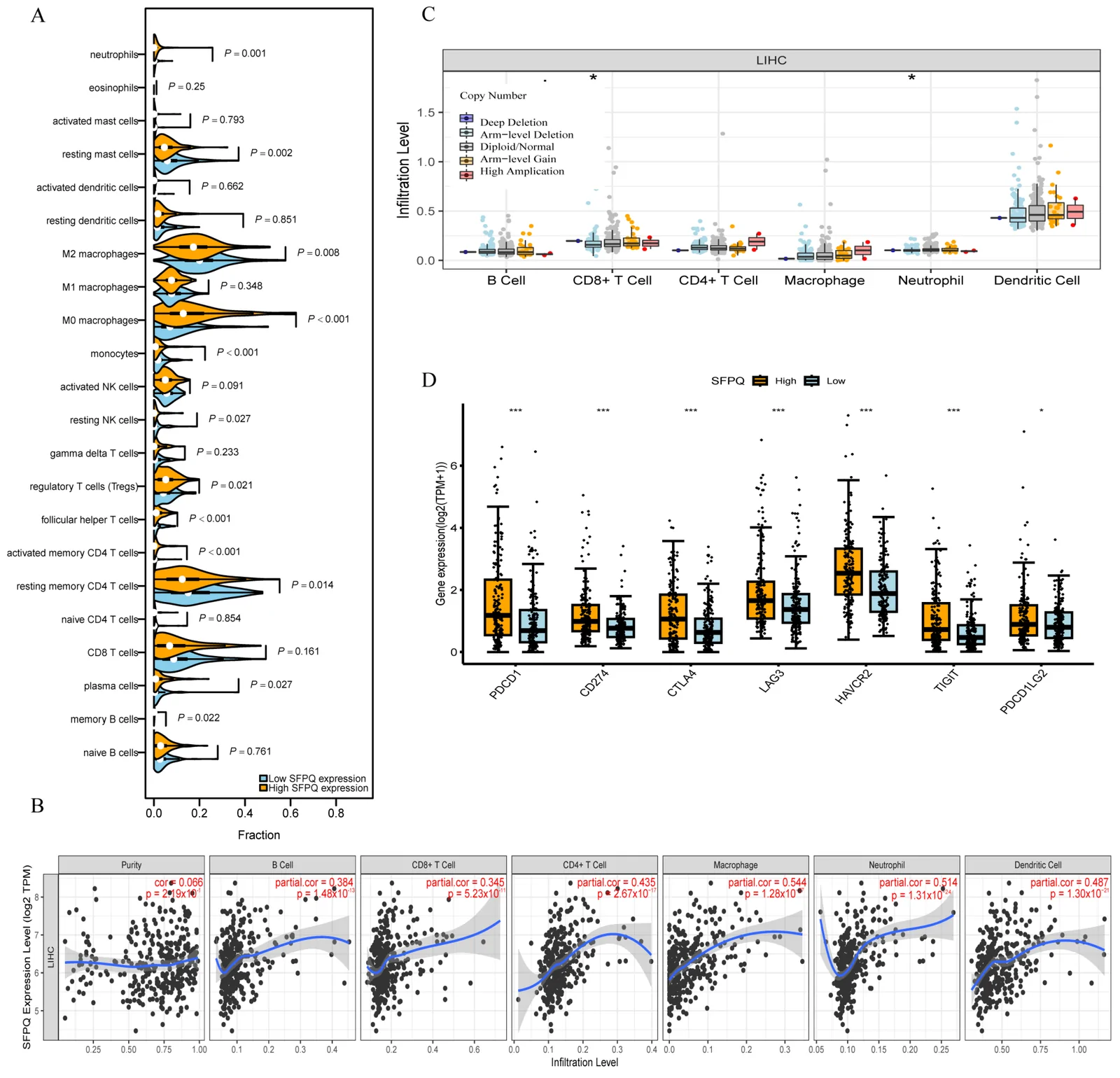
(**A**) The violin graph shows the difference in immune cells in the high- and low-SFPQ-expression groups. (**B**) The correlation between SFPQ expression and the degree of immune cell infiltration. The blue line represents the fitted curve of the correlation, and the grey shaded area indicates the 95% confidence interval. (**C**) Somatic copy number variations in the *SFPQ* gene are significantly associated with the infiltration levels of certain immune cells in HCC. (**D**) Differences in the proportions of 7 immune checkpoints in the HCC specimens in the high- and low-SFPQ-expression groups.* *p* < 0.05, *** *p* < 0.001.

Given that SFPQ is upregulated in HCC and strongly associated with poor prognosis, it may exert oncogenic functions in HCC. We therefore investigated whether SFPQ plays a pathogenic role in the HCC immune microenvironment. First, we evaluated the distribution of 22 tumor-infiltrating immune cell (TIIC) subtypes across different SFPQ expression groups based on the TCGA-LIHC cohort (Figure 4A,Table S5).

Our study revealed differences in immune cell infiltration abundance between the high- and low-SFPQ-expression groups, involving involving memory B cells, plasma cells, resting memory CD4 T cells, activated memory CD4 T cells, follicular helper T cells, regulatory T cells (Tregs), resting NK cells, monocytes, M0 macrophages, M2 macrophages, resting mast cells, and neutrophils. Among these, immune cells positively correlated with SFPQ expression included activated memory CD4 T cells, follicular helper T cells, regulatory T cells (Tregs), and M0 macrophages. In contrast, the infiltration levels of resting memory CD4 T cells, M2 macrophages, and resting mast cells were significantly lower in HCC patients with low SFPQ expression than in those with high SFPQ expression.

Subsequently, the TIMER method was used to evaluate the association between SFPQ expression and infiltrating immune cells in HCC (Figure 4B). SFPQ expression positively correlated with the infiltration levels of the following immune cells: B cells (r = 0.384, *p* = 1.48 × 10^−13^), CD8^+^ T cells (r = 0.345, *p* = 5.23 × 10^−11^), CD4^+^ T cells (r = 0.435, *p* = 2.67 × 10^−17^), macrophages (r = 0.544, *p* = 1.28 × 10^−27^), neutrophils (r = 0.514, *p* = 1.31 × 10^−24^), and dendritic cells (DCs, r = 0.487, *p* = 1.3 × 10^−21^). Conversely, SFPQ expression showed no significant association with tumor purity (*p* > 0.05). Additionally, our research assessed the impact of somatic copy number variation in SFPQ on the HCC immune microenvironment. This analysis revealed that somatic copy number variation in SFPQ is strongly correlated with immune infiltration by neutrophils and CD8^+^ T cells (Figure 4C).

It is well established that PD-1 (PDCD1), CTLA-4, TIM-3 (HAVCR2), LAG-3, PD-L1 (CD274), PDCD1LG2, and TIGIT serve as key inhibitory immune checkpoints implicated in immune escape within the tumor microenvironment. To further investigate this, the TCGA-LIHC cohort was utilized to evaluate the expression levels of these immune checkpoints across different SFPQ mRNA expression groups. The results demonstrated that the high-SFPQ-expression group exhibited significantly elevated levels of PDCD1, CD274, TIGIT, CTLA4, HAVCR2, LAG3, and PDCD1LG2 compared with the low-SFPQ-expression group (Figure 4D). Collectively, these findings suggest that SFPQ may modulate immune checkpoint-mediated immune responses in HCC, thereby influencing immune cell infiltration within the tumor microenvironment.

### 3.5. ASE Data Analysis and Screening of OS-SEs in HCC

**Figure 5:**
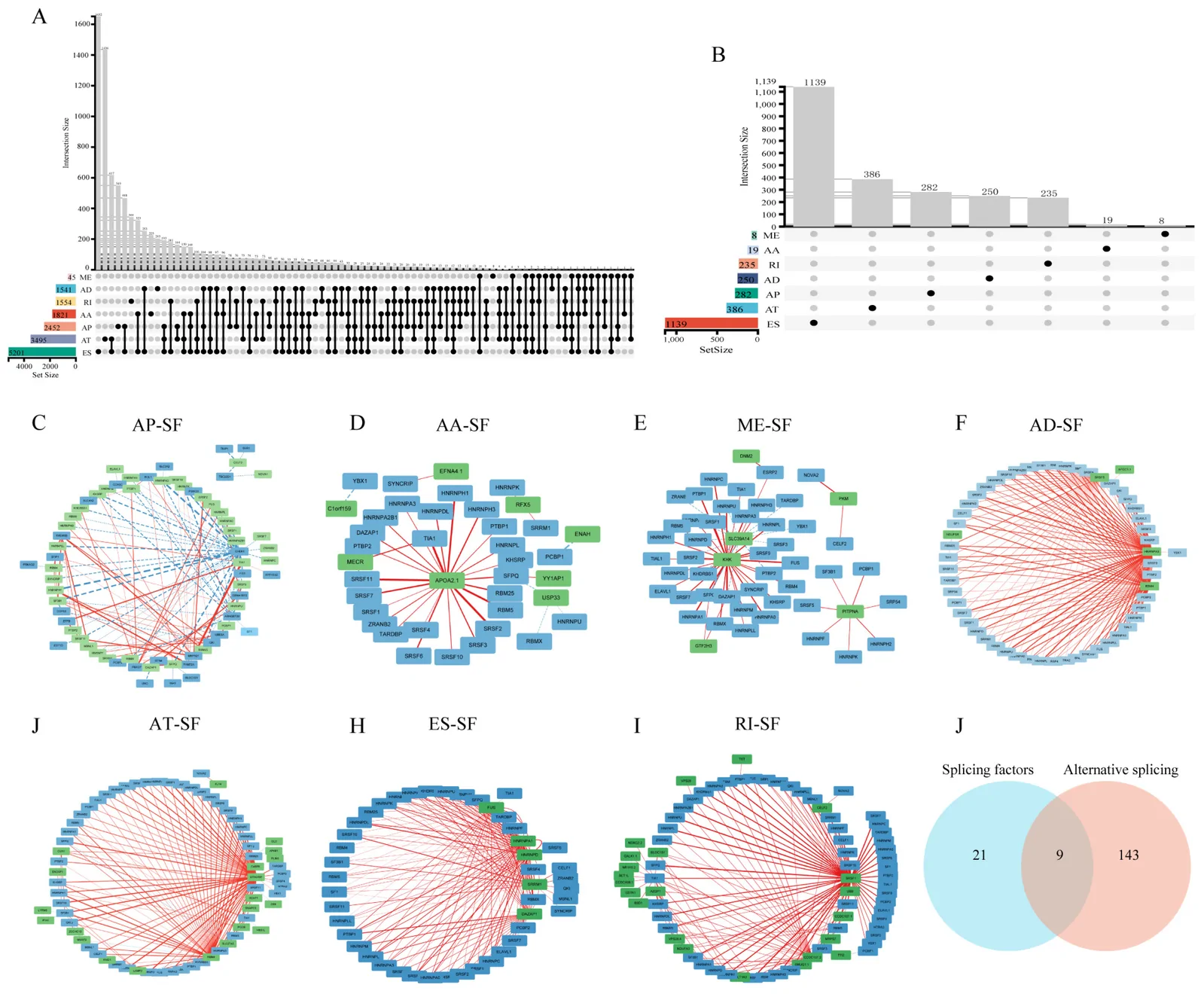
This study provides an overview of seven types of alternative splicing (AS). (**A**) UpSet plots displays the genes involved in the seven alternative splicing events and their mutual intersections in HCC. (**B**) Cox univariate analysis revealed seven survival-associated alternative splicing variants. (**C**–**I**) Construction of the interaction network of survival-related SFs and survival-related AS events to identify SF-associated ASEs. (**J**) The Venn diagram shows 30 genes of ASEs closely associated with five SFs, intersecting with 152 genes of ASEs closely associated with LASSO regression analysis.

SFs have been recognized as key players in the development and progression of HCC by regulating AS events in precursor mRNAs. To investigate this further, ASE data were downloaded from the TCGA-SpliceSeq database. The ASEs encompass seven types of variable splicing events: Alternate Acceptor site (AA), Alternate Donor site (AD), Alternate Promoter (AP), Alternate Terminator (AT), Exon Skip (ES), Mutually Exclusive Exons (ME), and Retained Intron (RI). A total of 21,919 ASEs were identified across 16,109 genes in the HCC cohort, comprising 2316 AA events in 1821 genes, 1908 AD events in 1541 genes, 9550 ES events in 5201 genes, 2153 RI events in 1554 genes, 2452 AP events in 2452 genes, 3495 AT events in 3495 genes, and 45 ME events in 45 genes. Moreover, multiple types of ASEs may occur within the same gene (Figure 5A). Univariate Cox regression analysis revealed 2319 AS events associated with overall survival (OS-ASEs), including 19 AA events, 250 AD events, 1139 ES events, 235 RI events, 282 AP events, 386 AT events, and 8 ME events (Figure 5B).

### 3.6. Network Construction of Regulatory Interactions Between ASEs and SFs

Given that SFs can influence ASEs, Spearman correlation analysis was performed between OS-SEs and OS-SFs to identify SF-associated ASEs. Subsequently, regulatory networks were constructed using Cytoscape software (Figure 5C–I). The results indicated that 61 OS-SFs were correlated with seven types of ASEs including 2319 AS events. A total of 30 ASEs were identified to be closely associated with five key SFs, including SFPQ, HTRA2, DAZAP1, PCBP2, and YBX1. LASSO regression analysis further identified major ASEs significantly associated with HCC prognosis, involving 152 genes across 167 ASEs: 92 AA events, 8 AD events, 27 ES events, 12 RI events, 9 AP events, 13 AT events, and 6 ME events. Integration of the above two analyses yielded nine ASEs (Figure 5J). The table shows the potential ASEs regulated by their corresponding SFs, with a single SF potentially regulating multiple splicing events (Table S6). SFPQ participates in regulating multiple alternative splicing events. Notably, SFPQ influences the ME event of GTF2H3 by regulating exon 10 or 11.

### 3.7. Correlation Analysis of GTF2H3 ME Events with HCC Survival and Clinical Parameters

**Figure 6:**
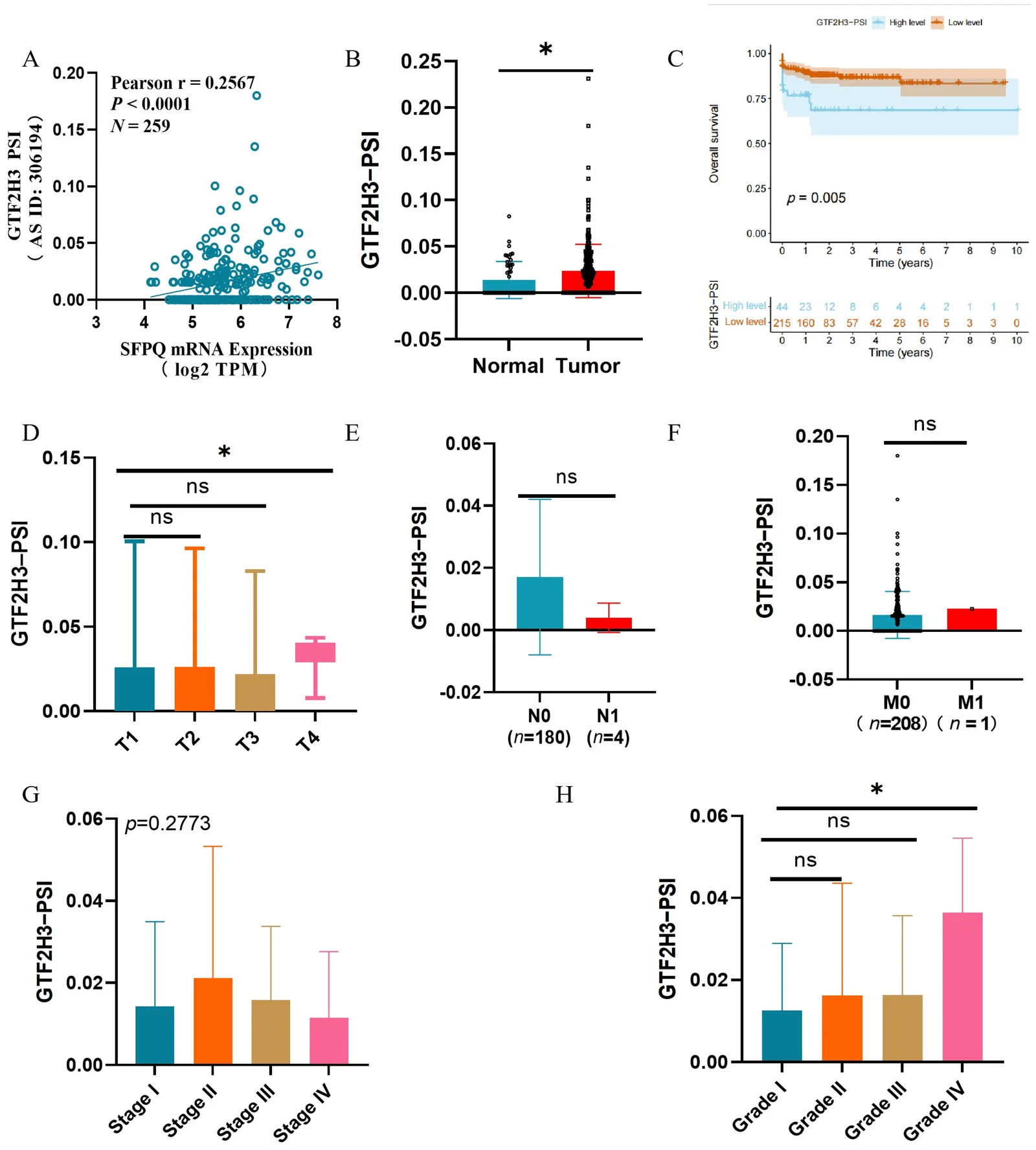
Correlation analysis of SFPQ expression and GTF2H3 PSI with clinical parameters in HCC. (**A**) Correlation plot showing a significant positive correlation between SFPQ mRNA expression and GTF2H3 PSI (Pearson r = 0.2567, *p* < 0.0001, *N* = 259). (**B**) GTF2H3 PSI was significantly higher in HCC tumor tissues compared to normal tissues. (**C**) Kaplan–Meier survival analysis showing that patients with higher GTF2H3 PSI values had a poorer overall survival (log-rank test, *p* = 0.005).(**D**−**G**) Boxplots showing the correlation between GTF2H3 PSI and clinicopathological parameters: (**D**) T stage (Kruskal–Wallis test); (**E**) N stage (Wilcoxon test); (**F**) M stage (Wilcoxon test); (**G**) pathological stage (Kruskal–Wallis test); (**H**) pathological grade (Kruskal–Wallis test). ns: not significant; * *p* < 0.05.

SFPQ influences the ME (exon 10/11) event of GTF2H3. To further validate this relationship, we first evaluated the correlation between SFPQ expression and the GTF2H3 PSI value in the TCGA-LIHC cohort. As shown in Figure 6A, SFPQ expression was significantly positively correlated with the GTF2H3 PSI value (Pearson r = 0.2567, *p* < 0.0001, *n* = 259). This association remained significant after FDR correction (FDR < 0.0001). Importantly, a partial correlation analysis adjusting for tumor stage and grade confirmed that the correlation persisted independently of these clinical variables (partial r = 0.256, *p* = 3.23 × 10^−5^).

Consequently, we investigated the correlation between GTF2H3 PSI and clinicopathological features in HCC patients (Figure 6B–H). GTF2H3 exhibited a significantly higher PSI in tumor tissues compared to normal tissues (Figure 6B). Moreover, a higher PSI was associated with poorer overall survival (Figure 6C). In the TNM staging system, the PSI for T4 was significantly higher than that for T1 (Figure 6D), while no significant differences were observed in the M, N, or other T stages (Figure 6E–G). Additionally, patients with grade IV tumors displayed a higher PSI than those with grade I tumors (Figure 6H). These findings suggest a positive correlation between the GTF2H3 exon 10/11 ME event and poor clinical prognosis in HCC.

### 3.8. The GTF2H3 ME Event Influences Cell Viability and Migration in HCC

**Figure 7:**
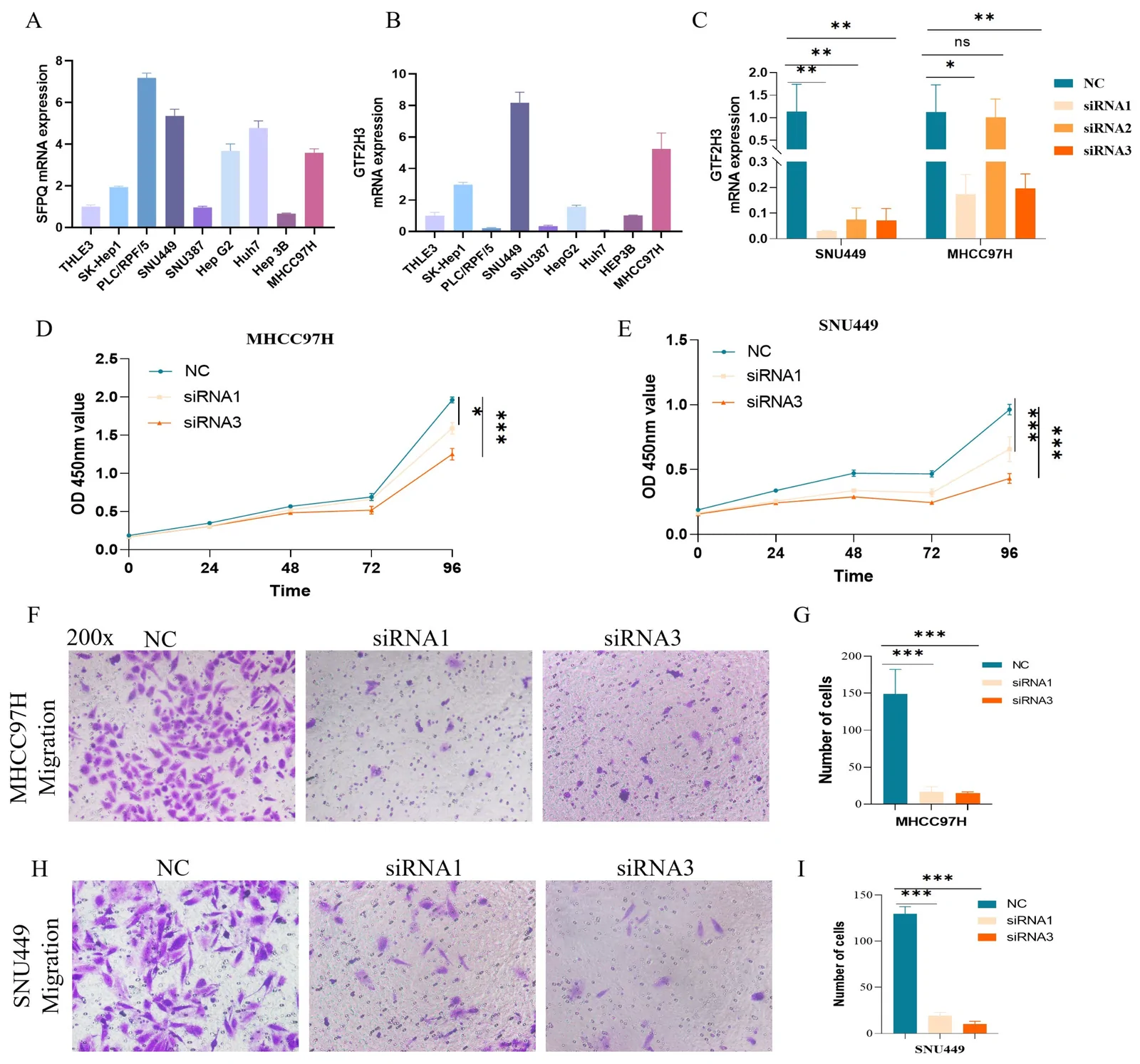
(**A**,**B**) SFPQ and GTF2H3 mRNA expression was detected by quantitative RT-qPCR in HCC cell lines and THLE3 cells. (**C**) GTF2H3 mRNA expression was detected by quantitative RT-qPCR after 48 h of transfection with NC siRNA or two different siRNAs targeting GTF2H3 (siRNA1, siRNA2, and siRNA3). (**D**,**E**) CCK-8 reagents were used to assay the viability ability at different time points after siRNA interference in HCC cells ((**C**) MHCC97H and (**D**) SNU449). (**E**,**G**) GTF2H3 induces HCC cell (MHCC97H and SNU449) migration. (**F**–**I**) The number of cells of GTF2H3-induced migration in HCC cells (MHCC97H and SNU449). Data are presented as the mean ± standard deviation (SD). ns: not significant; * *p* < 0.05, ** *p* < 0.01, and *** *p* < 0.001.

The expression levels of SFPQ and GTF2H3 were examined across in normal hepatocytes THLE3 and a panel of HCC cell lines, including SK-Hep1, PLC/PRF/5, SNU449, SNU387, HepG2, Huh7, HEP3B, and MHCC97H (Figure 7A,B). RT-qPCR analysis revealed that SFPQ and GTF2H3 are highly expressed in both MHCC97H and SNU449 cell lines. Consequently, these two cell lines were selected for subsequent siRNA-mediated knockdown experiments. Among the siRNAs tested, siRNA1 and siRNA3 effectively reduced GTF2H3 expression in MHCC97H and SNU449 cells compared with the negative control (NC) group (Figure 7C). Functional assays demonstrated that GTF2H3 knockdown significantly suppressed cell viability (Figure 7D,E). Moreover, migration assays indicated that GTF2H3 silencing markedly reduced the number of invasive cells in both cell lines relative to the NC group (Figure 7F–I).

**Figure 8:**
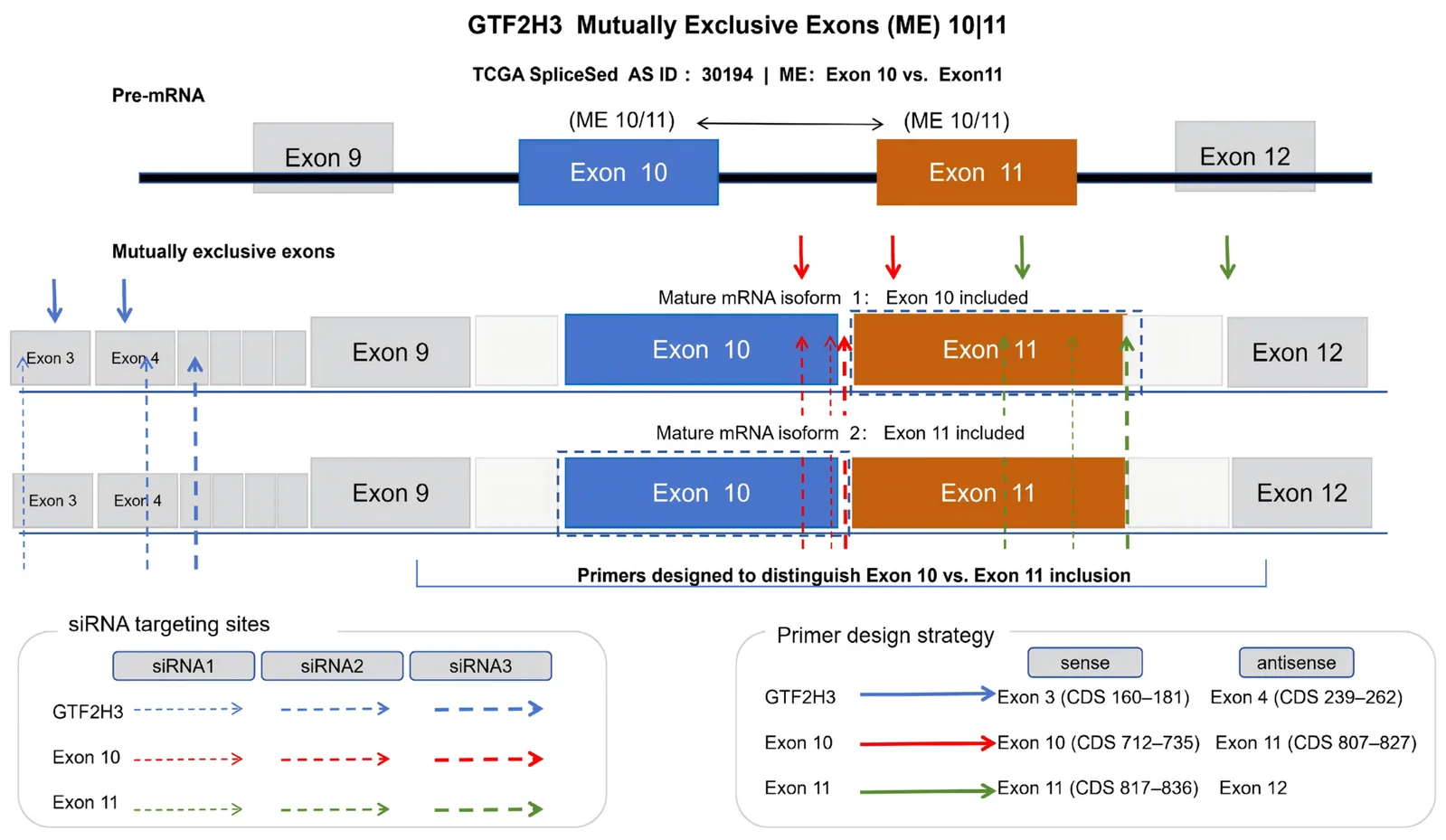
Schematic illustration of the GTF2H3 mutually exclusive (ME) exon 10/11 event and the design of siRNA and primers.

To further investigate the functional significance of the mutually exclusive exons 10 and 11 of GTF2H3, we designed specific siRNAs and primers to distinguish between the two isoforms. As illustrated in Figure 8, the siRNAs were designed to target either the internal sequences of exon 10 or exon 11 or the specific exon–exon junctions (e.g., exon 10/11 and exon 11/12 boundaries) to ensure selective knockdown. Concurrently, isoform-specific primers were designed to accurately quantify the inclusion of exon 10 or exon 11. Functional assays revealed that knockdown of GTF2H3 significantly reduced cell viability and migration in HCC cells.

**Figure 9:**
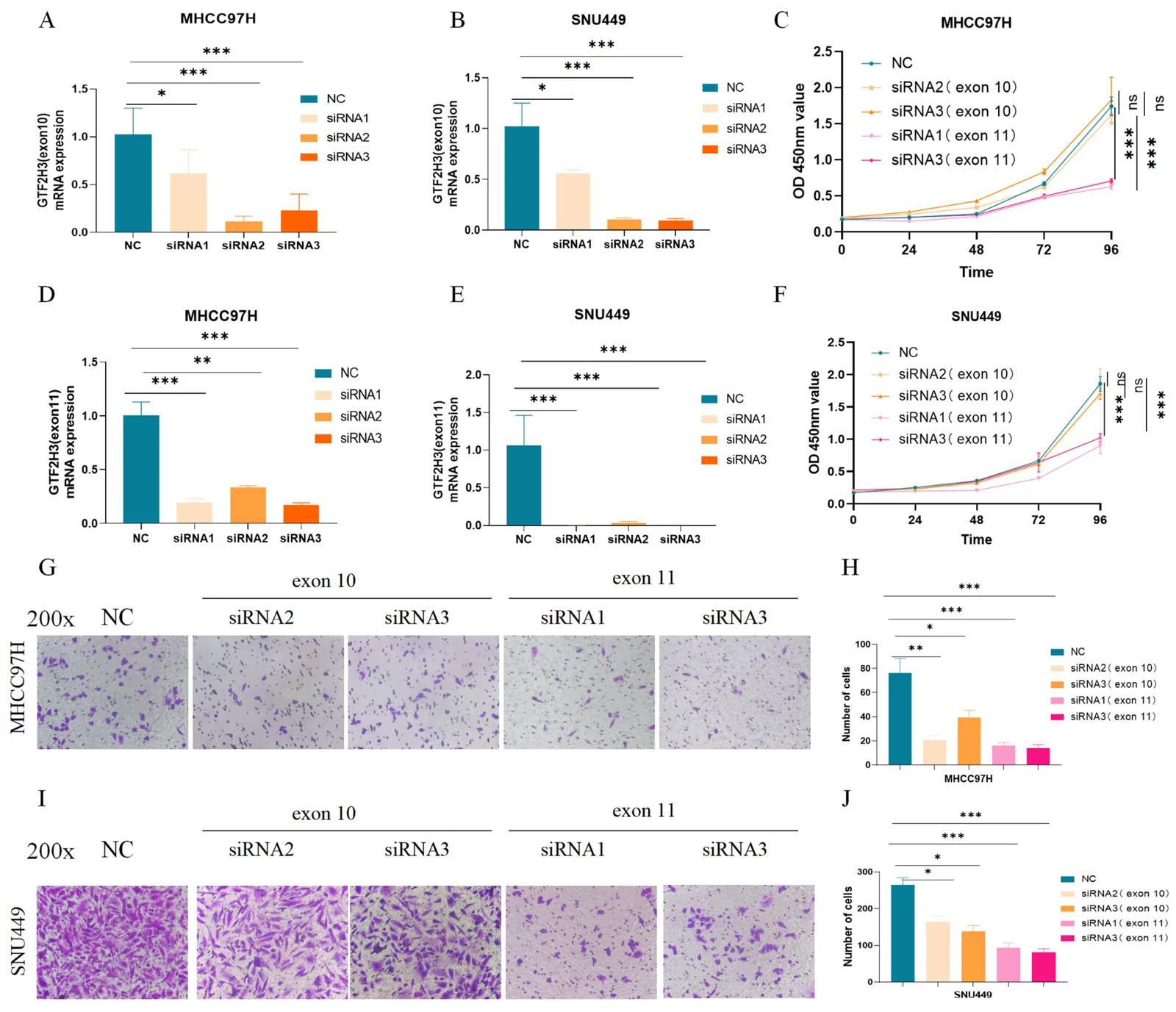
(**A**,**B**) mRNA expression was detected by quantitative RT-qPCR in MHCC97H via knockdown of exon 10 or exon 11. (**C**) CCK8 reagents were used to assay the cell viability at different time points after siRNA interference in MHCC97H cells. (**D**,**E**) mRNA expression was detected by quantitative RT-qPCR in SNU449 via knockdown of exon 10 or exon 11. (**F**) CCK8 reagents were used to assay the cell viability ability at different time points after siRNA interference in SNU449 cells. (**G**–**J**). Knocking down either exon 10 or 11 of GTF2H3 significantly suppressed the number of migrating cells in MHCC97H and SNU449 cell lines. In the CCK-8 experiments, statistical significance markers in the figures indicate the results of comparisons at the final time point. Data are presented as the mean ± standard deviation (SD). ns: not significant,* *p* < 0.05, ** *p* < 0.01, and *** *p* < 0.001.

Next, siRNAs targeting these exons were designed and subjected to functional assays (Figure 9). RT-qPCR screening identified siRNAs specific to GTF2H3 exons 10 (Figure 9A,D) and 11 (Figure 9B,E). Cell viability assays demonstrated that knockdown of exon 11 significantly suppressed the viability of MHCC97H and SNU449 cells, whereas knockdown of exon 10 exerted no notable effect (Figure 9C,F). Migration assays further revealed that silencing either exon reduced the number of migratory cells in both cell lines (Figure 9G–J), with exon 11 knockdown exhibiting a stronger inhibitory effect. Collectively, these findings suggest that GTF2H3 plays a pro-tumorigenic role in HCC.

## 4. Discussion

Our study systematically identified survival-associated SFs in HCC and preliminarily explored their functional and clinical significance. Leveraging Cox and LASSO regression analyses, we screened five key SFs—SFPQ, HTRA2, DAZAP1, PCBP2, and YBX1—from a pool of 71 candidates. These SFs demonstrated significantly elevated expression levels in HCC tissues relative to normal tissues, and their overexpression correlated strongly with poorer OS. Notably, multivariate analysis established SFPQ as an independent adverse prognostic factor for HCC; consequently, we selected SFPQ as a molecular target for subsequent in-depth investigation. This aligns with a previous study confirming that SFPQ promotes proliferation, migration, and invasion in HCC cells and is linked to poor prognosis [28]. Additionally, the clinical relevance of these SFs was underscored by their associations with an advanced T stage, higher tumor grade, and advanced clinical stage, consistent with prior reports linking SF dysregulation to aggressive cancer phenotypes. The observation that SFPQ, DAZAP1, PCBP2, and YBX1 correlated with the T stage, grade, and overall stage—while HTRA2 lacked a significant association with tumor grade—suggests distinct functional roles among these SFs, warranting further investigation into their context-dependent mechanisms.

Recent studies have highlighted SFPQ as a pivotal variable splicing factor in various tumors. In glioma cells, SFPQ directly participates in the splicing process of the SLC7A11 transcript, ensuring the retention of its terminal exon to generate mature SLC7A11 mRNA, thereby enhancing cellular resistance to ferroptosis inducers such as erastin and sulfasalazine (SAS). Loss of SFPQ function sensitizes cells to ferroptosis and impedes tumor growth [29]. Mechanistically, SFPQ is involved in the early stages of spliceosome assembly, influencing splice site selection through interactions with RNA and other splicing factors, such as U1-70K. Its phosphorylation state can modulate these interactions, thereby affecting splicing efficiency and the balance between exon inclusion and skipping [30]. In our study, functional enrichment analysis of SFPQ-associated splicing genes revealed their predominant involvement in key biological processes, including lipid metabolism, apoptosis, DNA damage response, and mRNA processing. This suggests that SFPQ may drive HCC progression by reprogramming cellular metabolism, stress response pathways, and mRNA processing. It is well established that metabolic reprogramming and immune evasion are two fundamental hallmarks of cancer. The metabolic environment can shape the liver’s immune response, thereby enabling tumor cells to evade immune surveillance.

A growing body of evidence suggests that both innate immune cells (such as macrophages, dendritic cells, natural killer cells, regulatory T cells, and neutrophils) and adaptive immune cells (including B cells and T cells) can reshape the tumor microenvironment, thereby promoting tumor growth, metastasis, and treatment resistance [31,32]. This interaction has also been demonstrated in HCC [33]. Based on our analysis of SFPQ expression, immune cell infiltration, and immune checkpoint inhibitors, we found that SFPQ expression is associated with immune cell infiltration. Specifically, the infiltration levels of several immune cell subsets positively correlated with SFPQ expression, including activated CD4^+^ memory T cells, follicular helper T cells, regulatory T cells (Tregs), and M0 macrophages. Conversely, in HCC samples with low SFPQ expression, the infiltration levels of quiescent CD4^+^ memory T cells and quiescent M2 macrophages were significantly lower than those in samples with high SFPQ expression. Therefore, we speculate that SFPQ may promote HCC progression by modulating the infiltration of tumor-associated immune cells. Within the tumor microenvironment, T lymphocytes play a key role in cell-mediated immunity by targeting tumor cells through tumor-specific antigens. Notably, immune checkpoint inhibitors can suppress T-cell activation and promote T-cell exhaustion [34].

SFPQ expression shows a potential association with immune checkpoint expression. Our study revealed that compared with the low-SFPQ-expression group, the high-SFPQ-expression group exhibited significantly elevated expression levels of PDCD1, CTLA4, TIGIT, LAG3, and TIM3. High expression of these genes in the TME is associated with a weakened T-cell-mediated anti-tumor immune response [35]. Among them, PD-1 (encoded by PDCD1) is a key regulator of effector T-cell immune responses, inducing T-cell exhaustion by delivering inhibitory signals to PD-1 expressing T cells [36]. CTLA4 is a critical inhibitor of T-cell proliferation and contributes to the formation of an immunosuppressive TME in HCC [37]. Notably, antibodies targeting PD-1/PD-L1 and CTLA-4 have been established as primary immunotherapeutic agents for various hematological and solid malignancies [38,39]. Overall, SFPQ expression is computationally associated with specific immune cell infiltration patterns and immune checkpoint expression, suggesting a potential link between SFPQ and the tumor immune microenvironment that warrants further investigation.

To elucidate the pathogenic mechanisms of these SFs, we constructed a regulatory network linking OS-SFs to OS-SEs. Our analysis identified SFPQ as a regulator of multiple alternative splicing events, including alternative promoter (AP) events (LAH1 and RCL1), alternative acceptor (AA) events (USP33, APOA2, and YY1AP1), and mutually exclusive exon (ME) events (GTF2H3, SLC39A14, and KHK). Notably, our study revealed that ME represents the rarest splicing type in HCC, accounting for only 0.2% of total events, with only eight ME events closely associated with HCC prognosis. Despite their low frequency, ME events can generate mRNA isoforms with distinct structural and functional attributes, potentially contributing to tumor heterogeneity. This heterogeneity may influence phenotypic outcomes such as cell proliferation and invasion and may also play a role in tumor immune regulation [40,41]. Consequently, we placed particular emphasis on investigating ME events regulated by SFPQ.

In the context of SFPQ-regulated mutually exclusive (ME) splicing events, we focused on its role in regulating the ME event of GTF2H3 by modulating the inclusion of exon 10 or 11. The general transcription factor IIH subunit 3 (GTF2H3) gene encodes a member of the TFB4 family, and the encoded protein is a subunit of the general transcription factor IIH complex (TFIIH) [42]. TFIIH plays a central role in transcription initiation and promotes transcription elongation by phosphorylating the C-terminal domain (CTD) of RNA polymerase II [43]. As a TFIIH subunit, GTF2H3 may indirectly contribute to the maintenance of drug-resistant phenotypes by regulating gene transcription, thereby activating pathways involved in cell cycle progression and survival signaling. Furthermore, GTF2H3 may synergistically enhance DNA repair and transcription efficiency by stabilizing the TFIIH complex structure or modulating its enzymatic activity, thus jointly promoting the survival of drug-resistant cells [44]. A previous study identified a fusion between the *GTF2H3* gene and the *SETD1B* gene in patients with polycythemia vera, resulting in the *5′-SETD1B*/*3′-GTF2H3* fusion gene. The specific breakpoints occur at intron 11 of SETD1B and intron 5 of GTF2H3. Following fusion, the transcript exhibits a frameshift connection between exon 11 of SETD1B and exon 7 of GTF2H3 [45]. To date, the role of GTF2H3 in HCC has not been investigated.

We further evaluated the correlation between the PSI value of GTF2H3 and the clinicopathological characteristics of patients with HCC. Compared with normal tissues, HCC tumor tissues exhibited higher PSI values of GTF2H3, and patients with elevated PSI values had a poorer prognosis. Therefore, we hypothesize that the ME event involving exons 10 and 11 of GTF2H3 is positively associated with poor clinical outcomes in HCC. Functional assays revealed that knockdown of GTF2H3 significantly reduced cell viability and migration in HCC cells, suggesting that the GTF2H3 ME event may play a tumor-promoting role. We then designed siRNAs specifically targeting exon 10 or exon 11 of GTF2H3. Knockdown of exon 11 significantly reduced the survival rates of MHCC97H and SNU449 cells. In migration assays, knockdown of either exon 10 or exon 11 of GTF2H3 significantly reduced the number of migrating cells in both cell lines compared with the NC group. Notably, the inhibitory effect on cell migration was more pronounced following exon 11 knockdown than following exon 10 knockdown. However, further protein-level and phenotypic validation is required to confirm these findings. Collectively, these data indicate that the ME event of GTF2H3 plays a tumor-promoting role in HCC.

Building on the functional link between SFPQ and GTF2H3 observed in our study, we hypothesize that SFPQ may regulate this oncogenic GTF2H3 ME event. However, a limitation of our study is that we did not perform RNA immunoprecipitation (RIP)-qPCR to directly confirm their physical interaction. Therefore, the direct molecular binding of SFPQ to GTF2H3 pre-mRNA remains a hypothesis. Future studies employing RIP-qPCR or comparable RNA-binding assays are warranted to validate this mechanism.

## 5. Conclusions

In summary, our study identifies SFPQ as a key survival-related splicing factor in HCC, with overexpression associated with aggressive clinicopathological features and poor prognosis. SFPQ exerts its oncogenic effects through mechanisms involving the regulation of specific splice variants (e.g.,), transcripts containing GTF2H3 ME) and the modulation of immune cell infiltration and checkpoint expression. These findings highlight SFPQ and its downstream targets (e.g.,GTF2H3) as promising biomarkers and therapeutic candidates for HCC. Further studies are needed to validate these mechanisms in preclinical models, including splice-switching and isoform-specific rescue approaches, and to explore SFPQ-targeted strategies to improve HCC patient outcomes.

## Figures and Tables

**Table 1:** The sequences of siRNA.

siRNA	Sequences
GTF2H3-siRNA1	sense: GAAUCGUUCCAACAAACUU
	antisense: AAGUUUGUUGGAACGAUUC
GTF2H3-siRNA2	sense: CGUGCGAGACAGCCUUUAA
	antisense: UUAAAGGCUGUCUCGCACG
GTF2H3-siRNA3	sense: CAAGGAUAUUGGUGAUUAA
	antisense: UUAAUCACCAAUAUCCUUG
GTF2H3-exon10-1	sense: CGGGAGGACUGUACCUGAA
	antisense: UUCAGGUACAGUCCUCCCG
GTF2H3-exon10-2	sense: GCCUCAGAUGCCUUCUCUU
	antisense: AAGAGAAGGCAUCUGAGGC
GTF2H3-exon10-3	sense: CCUUCUCUUCUGCAGUAUU
	antisense: AAUACUGCAGAAGAGAAGG
GTF2H3-exon11-1	sense: GAUCAGAGAUCUCAGUUAA
	antisense: UUAACUGAGAUCUCUGAUC
GTF2H3-exon11-2	sense: CGAUCAAGAUCAGAGAUCU
	antisense: AGAUCUCUGAUCUUGAUCG
GTF2H3-exon11-3	sense: GGUUAUGUCUGUUCUGUGU
	antisense: ACACAGAACAGACAUAACC

**Table 2:** The sequences of primers.

Primers	Sequences
GTF2H3	sense: CCGTGATGGTGCTGGGAAATTC
	antisense: TCTCCGAAGAAGTCTCCAAGTCTG
GTF2H3-exon10	sense: AAAGACAATCAGGAAATGAAATC
	antisense: TGAGGCACCTTCAGGTACAGT
GTF2H3-exon11	sense: GTTGACTACAGGGCTGCTTG
	antisense: TTCCCAACAAAGAATCTGCTATA
β-actin	sense: CTCTTCCAGCCTTCCTTCCT
	antisense: AGCACTGTGTTGGCGTACAG

## Data Availability

The data analyzed and utilized in this study will be provided by the corresponding author upon reasonable request.

## Supplementary Materials

The following supporting information can be downloaded at: https://www.mdpi.com/article/10.3390/cancers18203258/s1, Figure S1: The expression levels of the key SFs in HCC across ICGC-LIRI-JP (with tumor and adjacent specimens). (A–E) Unpaired analyses show the mRNA expressions of the key SFs in HCC and adjacent samples. (F–J) Paired analysis shows the mRNA expression levels of these key SFs in HCC and adjacent samples. (** *p* < 0.01 and *** *p* < 0.001). Figure S2: (A–E) The overall survival (OS) prognostic value of the key SFs in HCC (Kaplan–Meier plotter) in the ICGC-LIRI-JP cohort. Figure S3. The prognostic value of the five SFs in HCC (Kaplan–Meier plotter), including progression-free survival (PFS), recurrence-free survival (RFS), and disease-specific survival (DSS): A. SFPQ, B. HTRA2, C. DAZAP1, D. PCBP2, and E. YBX1. Figure S4: (A) The association between SFPQ and AS events. (B–C) GO and KEGG analyses identified 385 genes associated with AS events related to SFPQ expression. (B) GO; (C) KEGG pathway. Table S1: Predicted target regions of GTF2H3 siRNAs within the coding sequence (CDS). Table S2: Seventy-one splicing factors (SFs). Table S3: Fifty splicing factors were significantly associated with patient prognosis. Table S4: Schoenfeld residuals test for the proportional-hazards assumption. Table S5: Differential abundance of 22 tumor-infiltrating immune cell (TIIC) subtypes between high and low SFPQ expression groups in the TCGA-LIHC cohort. Table S6. ASEs associated with five key SFs.

## Abbreviations

HCC	Hepatocellular Carcinoma
AS	Alternative Splicing
PSI	Percent Spliced in Index
SFs	Splicing Factors
SFPQ	Splicing Factor Proline and Glutamine Rich
TFIIH	Transcription Factor IIH Complex
GTF2H3	General Transcription Factor IIH Subunit 3
AA	Alternate Acceptor Site
AD	Alternate Donor Site
AP	Alternate Promoter
AT	Alternate Terminator
ES	Exon Skip
ME	Mutually Exclusive Exons
RI	Retained Intron
