# Supplementary material for: SFPQ Promotes Hepatocellular Carcinoma Progression by Affecting GTF2H3 Splicing Variants
Source: Cancers (Basel). 2026 Oct 9;18(20):3258. doi: 10.3390/cancers18203258 (PMC13702749; doi:10.3390/cancers18203258)
Supplement: Supplementary file 1 [file cancers-18-03258-s001.zip › FigureS-legend.pdf]

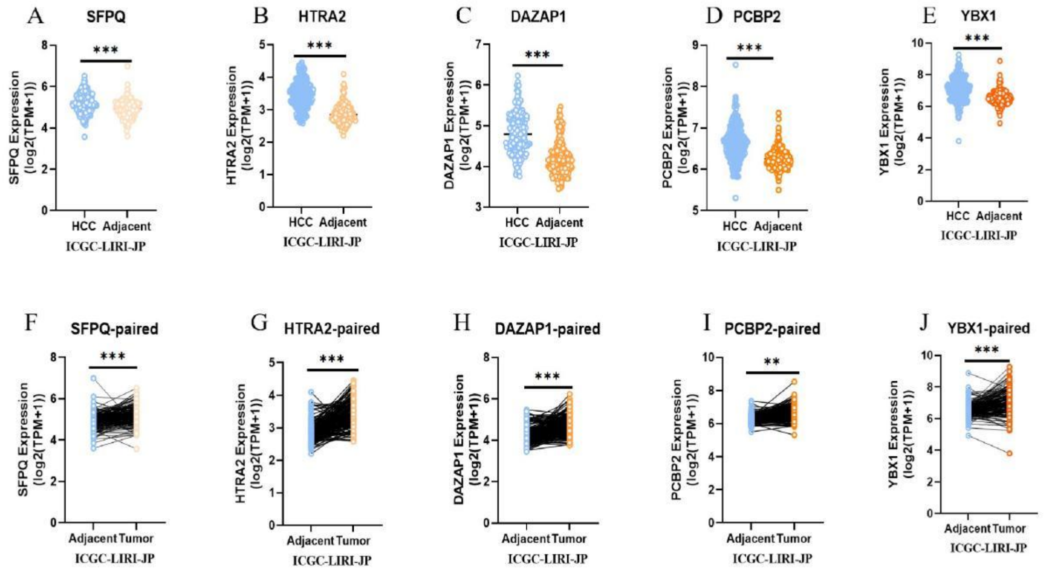

Figure S1: The expression levels of these key SFs in HCC across ICGC-LIRI-JP (with tumor and adjacent specimens).A-E.Unpaired analyses showed the mRNA expression of these key SFs in HCC and adjacent samples. F-J. Paired analysis showed the mRNA expression levels these key SFs in HCC and adjacent samples.(**$p < 0.01$ and *** $p < 0.001$)

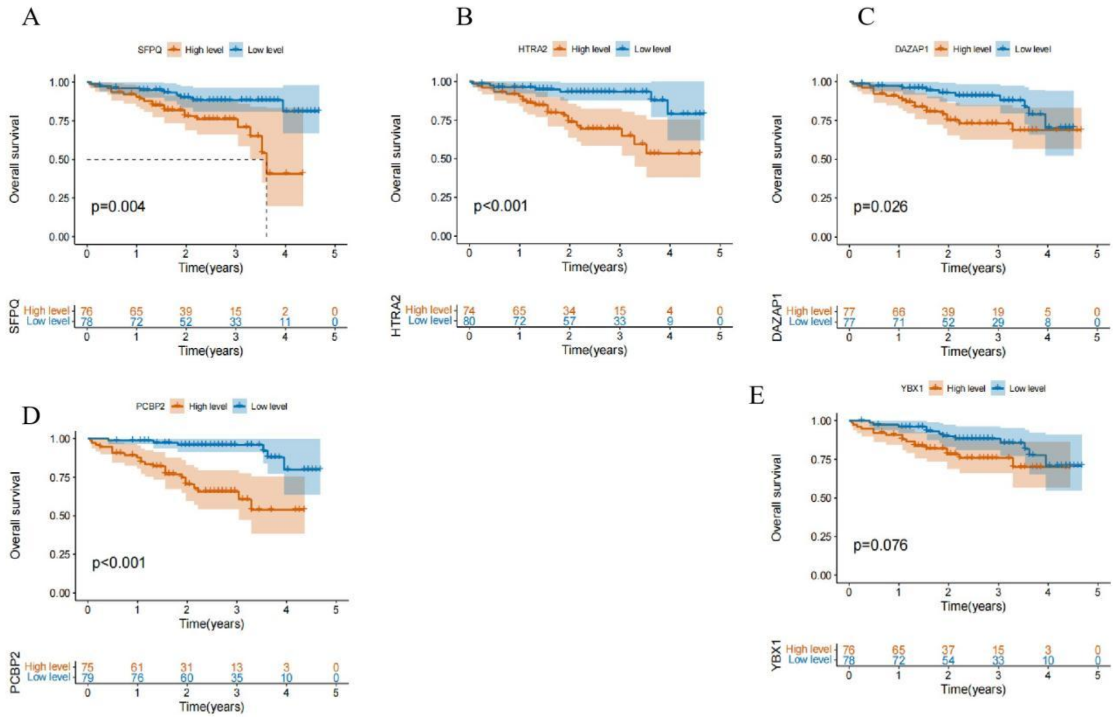

Figure S2: A-E.The overall survival prognostic value of these key SFs in HCC (Kaplan-Meier plotter) in ICGC-LIRI-JP cohort.

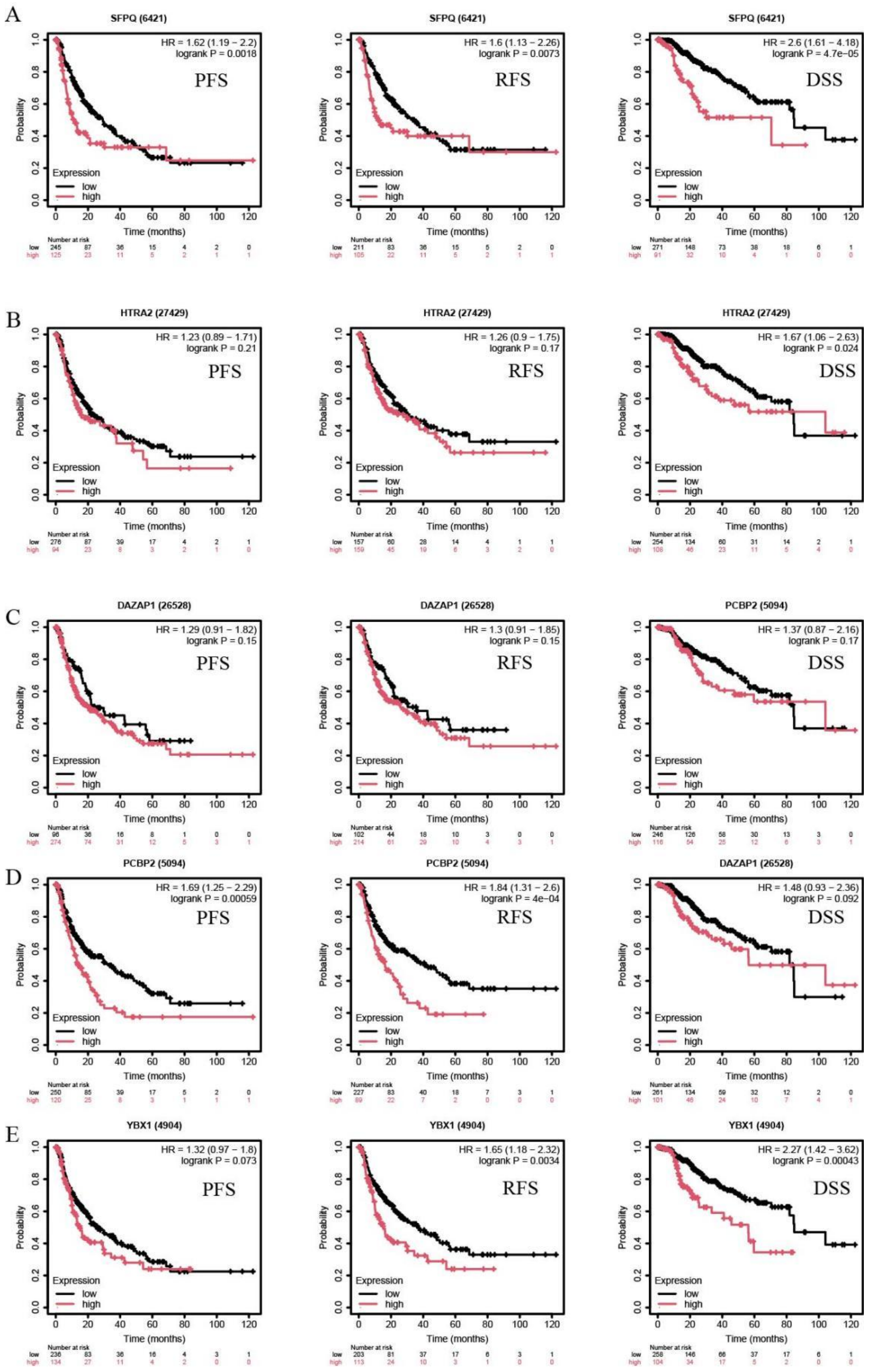

Figure S3.The prognostic value of the five SFs in HCC (Kaplan-Meier plotter) including progression free survival (PFS), recurrence free survival (RFS),disease specific survival (DSS),A.SFPQ,B.HTRA2,C.DAZAP1,D.PCBP2,E.YBX1.

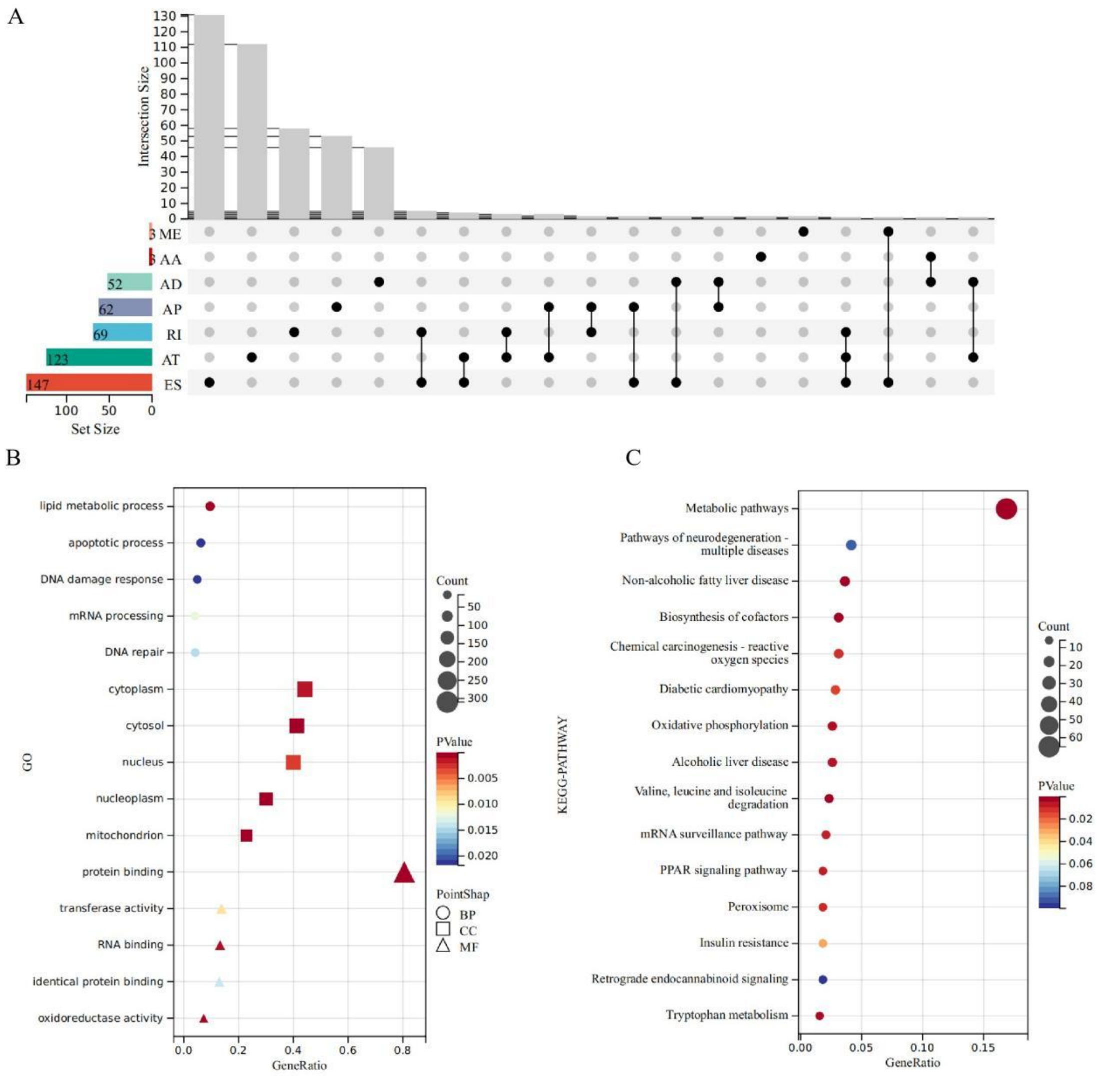

Figure S4. A.The association between SFPQ and AS events,B-C.GO and KEGG analyses identified 385 genes associated with AS events related to SFPQ expression.B.GO,C.KEGG pathway.
